# Multifactorial Causal Analysis of Workplace Musculoskeletal Disorders (WMSDs) and Psychological Stress Among Teaching Professionals for Adult Learners: A Narrative Review

**DOI:** 10.3390/healthcare13222897

**Published:** 2025-11-13

**Authors:** Kizhakematumal Jijo Alex, Faris Abdullah, Mohd Hafiidz Jaafar, Mark Harris Zuknik, Norhaniza Amil, Zitty Sarah Ismail

**Affiliations:** 1 School of Industrial Technology, Universiti Sains Malaysia, Gelugor 11900, Pulau Pinang, Malaysia; kjijo.alex@student.usm.my (K.J.A.);; 2 Occupational Safety and Health Unit, Engineering Campus, Universiti Sains Malaysia, Nibong Tebal 14300, Pulau Pinang, Malaysia; faris_abdullah@usm.my; 3 Faculty of Applied Sciences, Universiti Teknologi MARA, Shah Alam 40450, Selangor Darul Ehsan, Malaysia

**Keywords:** occupational ergonomics, workplace safety and health, work-related musculoskeletal disorder, stress, teaching professionals, adult learners

## Abstract

Work-related musculoskeletal disorders (WMSDs) and psychological stress remain major occupational health challenges among teaching professionals in adult education, yet their interconnected causes are often underexplored. This narrative review aims to identify multifactorial risk factors that contribute to these conditions and to propose a comprehensive framework that enhances understanding of teaching professionals’ well-being. A systematic synthesis of recent epidemiological and occupational health studies was conducted to analyse both immediate and underlying determinants across human, workplace, organisational, and socioeconomic dimensions. The findings reveal that more than two-thirds of teaching professionals experience WMSDs, particularly in the neck and lower back, while psychological stress affects over seventy percent globally. The combined effects of poor ergonomics, prolonged static postures, excessive workload, and limited organisational support contribute significantly to both physical and psychological strain. Broader contextual influences such as job insecurity, insufficient institutional resources, and societal undervaluation further intensify these risks. The review identifies a reciprocal relationship between physical discomfort and psychological distress, where each condition amplifies the other through behavioural and physiological mechanisms. The proposed integrative framework establishes a foundation for targeted interventions and evidence-based policy, promoting a shift toward holistic, system-oriented approaches to occupational health for teaching professionals in professional education settings.

## 1. Introduction

Teaching professionals in adult education face increasing occupational health risks, particularly WMSDs and psychological stress. Epidemiological data show that over 60% of teaching professionals experience WMSDs, most commonly neck and lower back pain, while more than 70% report psychological distress. These issues stem from a combination of poor ergonomics, prolonged static postures, repetitive tasks, and psychosocial stressors such as excessive workload, low social support, and limited job control. Teaching professionals for adult learners are uniquely burdened by the need to balance physical strain with complex cognitive and emotional labour, intensifying the bidirectional relationship between physical pain and psychological stress.

Despite growing awareness, current research often treats WMSDs and psychological stress as separate phenomena or lacks a comprehensive framework to explain their interdependence. This review addresses that gap by integrating three theoretical models to construct a multifactorial understanding of these occupational health challenges. The biopsychosocial model [1] provides a holistic lens to examine how biological, psychological, and social factors interact to influence health outcomes. Stock’s organisational model (2013) highlights how workplace structures, physical constraints, and psychosocial pressures contribute to both WMSDs and psychological distress. The Effort-Reward Imbalance (ERI) model [2,3] further explains how disproportionate work effort and insufficient rewards—such as recognition, job security, and career progression—can lead to negative emotional and physical health outcomes.

This review synthesises current evidence using an integrative framework that unites both linear and non-linear accident causation perspectives, drawing from the principles of Reason’s Organisational Accident Model, Rasmussen’s Risk Management Framework, Stock’s Organisational Model, the Systems-Theoretic Accident Model and Process (STAMP), and the ERI model. The framework conceptualises how systemic, organisational, and human factors dynamically interact to influence the occurrence of work-related musculoskeletal disorders (WMSDs) and psychological distress among teaching professionals for adult learners. The linear elements provide clarity on sequential cause–effect relationships, while the non-linear dimensions emphasise the complexity of multilevel and reciprocal feedback processes across human, organisational, and environmental systems.

**Figure 1:**
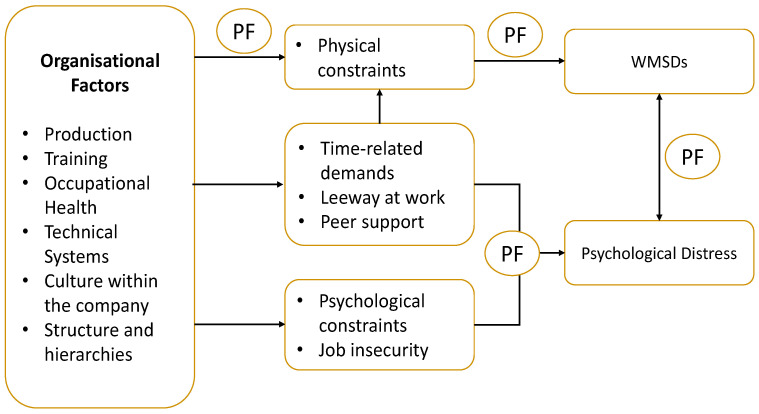
Stock Model (Roquelaure, 2018 [4]).

Although numerous studies have examined the prevalence and determinants of WMSDs and psychological stress among teaching professionals, limited attention has been directed toward teaching professionals for adult learners. Existing research has largely focused on school and university teaching professionals, with insufficient emphasis on the unique ergonomic and psychosocial challenges encountered by adult teaching professionals, including heterogeneous learner needs, irregular teaching schedules, and hybrid instructional modes. It has been suggested that musculoskeletal and psychosocial risks among teaching professionals are multifactorial and arise from complex interactions between individual, organisational, and societal dimensions, emphasising the need for integrative analytical approaches, as shown in Figure 1 [4].

In the context of this review, teaching professionals can be defined as educators that engaged in diverse pedagogical, organisational, and psychosocial roles who facilitate learning across formal and non-formal settings, while managing complex cognitive, emotional, and physical demands that influence their occupational health and well-being [5,6,7,8,9] for adult learning (focusing on professional trainings). Unlike primary, secondary or university faculty, adult-education teaching professionals face heterogeneous learners, irregular schedules, and hybrid learning environments, creating unique ergonomic and psychosocial pressures that remain underexplored.

The main objective of this study is to refine and validate a component of the proposed framework, thereby facilitating a thorough analysis of the inter-relationships, both direct and underlying, among these variables and their cumulative impact on the prevalence of WMSDs and psychological stress in the academic profession.

## 2. Method

This narrative review employed a flexible, integrative approach to synthesise existing literature on WMSDs and psychological stress experienced by teaching professionals working with adult learners. Unlike systematic reviews, this method prioritises thematic exploration and critical interpretation of research findings rather than exhaustive study identification. The focus was on capturing the multifactorial nature of these health issues by examining human, workplace, organisational, socioeconomic, and external stakeholder determinants, which further theoretically integrated into existing model.

### 2.1. Timeframe Covered by the Search

The majority of literature search (73%) covered studies published between January 2019 and June 2025, ensuring inclusion of both seminal and current research. However, some sources older than five years were also considered due to their continued relevance to contemporary studies, allowing for a comparison of conditions before the pandemic. These include foundational works that have shaped current research in the field.

### 2.2. Search Strategy, Including Terms and Boolean Combinations

Search terms were developed iteratively. Key terms and Boolean combinations included:

- “musculoskeletal disorders” OR “WMSDs” AND “teaching professionals”;
- “psychological stress” AND “adult learners”;
- “occupational health” OR “workplace health” AND (“ergonomics” OR “job control”).

Boolean operators (AND, OR) were used to expand (OR) or narrow (AND) the results. Truncation and phrase searching ensured comprehensive coverage.

### 2.3. Inclusion and Exclusion Criteria

Articles were included if they met the following inclusion criteria.

Inclusion criteria:

- Peer-reviewed articles in English and available in full text.
- Original research, reviews, or significant conceptual papers.
- Studies focused on musculoskeletal and psychological health in teaching professionals for adult learners and studies on primary/secondary teaching professionals.

Exclusion criteria:

- Non-peer-reviewed sources, opinion pieces, editorials, or conference abstracts or not available in full text.
- Non-teaching populations.
- Non-English publications.

### 2.4. Study Selection Process

**Figure 2:**
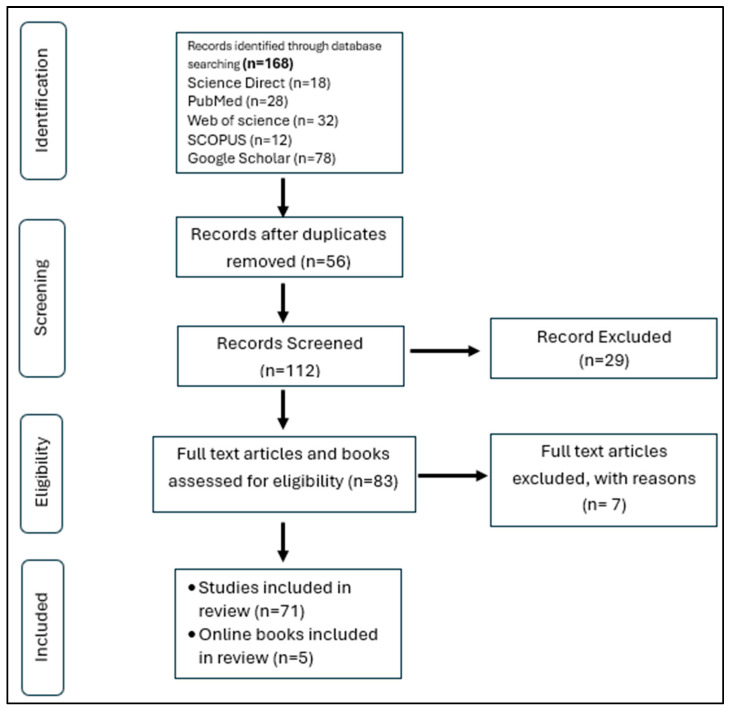
Flow chart of study selection in narrative review.

The database searches yielded a total of 168 entries (Figure 2). Of these, 56 duplicates were removed using “Zotero Version 5.0.188”. Of the remaining 112 studies screened via titles and abstracts, 83 studies met the inclusion criteria, and full texts were screened for final inclusion in the review. Reasons for exclusion included non-teaching professionals, the focus not being aligned with the current study, studies that were reviews, and studies that were not available in full text. Following the full-text screening, 7 papers, in total, were eliminated, leaving 76 studies eligible to move forward with the data extraction and narrative synthesis.

### 2.5. A Detailed Outlining the Search Strategy for Each Database

A comprehensive literature search was conducted using five major academic databases: ScienceDirect, PubMed, Scopus, Web of Science, and Google Scholar. The search covered the period from October 2023 to June 2025 to capture relevant foundational and recent studies. The full search strategy combined keywords and Boolean operators to maximise retrieval, including terms such as “musculoskeletal disorders” OR “WMSDs” AND “teaching professionals,” and “psychological stress” AND “adult learners.” Truncation and phrase searching were applied to ensure inclusiveness. The detailed search strings for each database are provided in Table 1.

During the review process, various factors (variables) contributing to WMSDs and psychological stress were examined to understand their impact on teaching professionals. Data from selected studies were extracted and synthesised thematically, focusing on the mechanisms and interplay of human, workplace, organisational, socioeconomic, and external stakeholder factors. This approach was employed, allowing for the integration of diverse review designs and methodologies.

## 3. Causes Contributing to WMSDs and Psychological Stress

WMSDs and psychological stress are two major health concerns that significantly impact the well-being and performance of teaching professionals. As teaching professionals face a unique set of challenges in their work environment, a combination of physical, emotional, and psychological factors contributes to the onset and escalation of these conditions. From poor ergonomics and repetitive movements to the pressures of managing classrooms and meeting high expectations, the causes of WMSDs and psychological stress are complex and multifaceted [10,11]. These health issues not only affect teaching professionals’ physical comfort but also have profound implications for their mental health, job satisfaction, and overall quality of life [5]. Understanding the root causes of these conditions is crucial to developing effective strategies to support teaching professionals and create healthier, more sustainable work environments. This section delves into the various factors contributing to WMSDs and psychological stress, shedding light on the intricate relationship between physical strain and mental well-being within the teaching profession.

**Figure 3:**
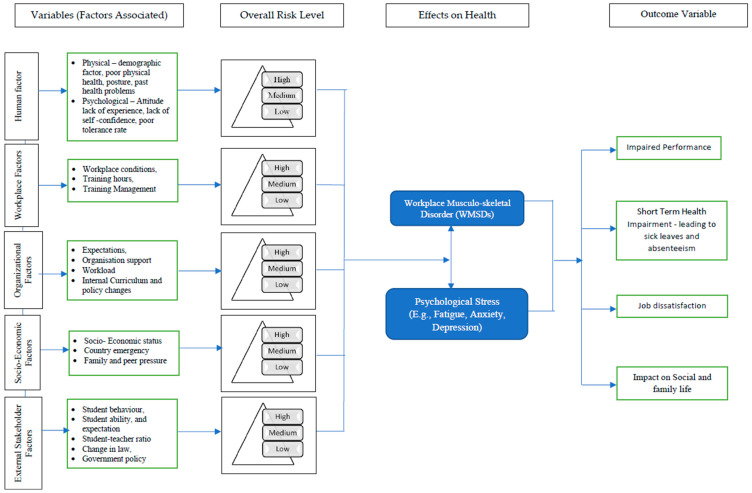
Conceptual framework on causes of WMSDs and psychological stress and its outcome.

As discussed earlier, different research articles were studied, and a new framework was proposed for WMSDs and psychological stress, as provided in Figure 3. The framework considers various factors acting as independent variables resulting in WMSDs, and psychological stress is presented in this section, outlining the basis of this review.

The conceptual framework elucidates the complex interrelationships among the multifarious determinants that collectively influence the incidence of WMSDs and psychological stress. Human factors are delineated into two principal subcategories: physical factors such as age, body weight, and pre-existing health conditions and psychological factors, encompassing individual attitudes, stress tolerance, and emotional resilience. Workplace factors are characterised by the physical and operational environment, including ergonomic conditions, hours dedicated to training, and the efficacy of training management protocols. Organisational factors comprise the expectations set by management, the availability and quality of institutional support, workload intensity, as well as internal curriculum and policy modifications. Socioeconomic factors extend to broader contextual influences, such as socioeconomic status, national or regional emergencies, and pressures emanating from family and peer groups. Finally, external stakeholder factors are operationalised through student behaviours, the clarity and comprehension of academic expectations, frequency of complaints, and student-teacher ratios, each of which can significantly impact the professional milieu and, consequently, teaching professionals’ well-being. This comprehensive framework thus integrates a spectrum of variables at multiple levels, facilitating a nuanced understanding of their synergistic effects on WMSDs and psychological stress among teaching professionals.

Human error, as the primary independent variable, is widely recognised as a leading contributor to workplace accidents and incidents. This phenomenon is influenced by a constellation of interrelated factors, including but not limited to age, sex, underlying health conditions, suboptimal work posture, communication breakdowns, coordination and cooperation failures, interpersonal conflicts, deficits in skills and knowledge, and attitudinal issues, all of which collectively heighten the risk of occupational accidents [12,13]. These same factors are also implicated in the development of WMSDs and psychological stress, which can culminate in chronic and long-term health impairments. In one study, risk factors associated with teaching professionals can arise from physical factors such as age, obesity, and gender [14]. The impact of gender on teaching professionals was observed in many studies. One such study [15] showed depression is twice as common in women teaching professionals compared to males. With the previous COVID situation, the pandemic can also psychologically impact the teaching professionals and generate fear, distress, and anxiety [7,16].

Furthermore, several studies on WMSDs affecting people working from home [17] have been conducted during the pandemic period. The shift from face-to-face to online teaching has caused significant distress among teaching professionals. Training online may lead to teaching professionals sitting for long hours glued to the screen, which can affect students mentally and physically. Prolonged sitting can lead to muscle fatigue, create muscular discomfort, and impair cognitive functions [18]. Apart from WMSDs, different demographic variables have different stressors [19]. Sex, age, years spent on the job and awkward postures have a high prevalence of WMSDs [20]. Human errors are subjective, vary from teacher to teacher, and can be complex due to multiple factors, as discussed earlier.

Workplace factors represent a critical independent variable in the aetiology of both physical and psychological stress among teaching professionals. These encompass a broad spectrum of environmental and organisational elements, including the adequacy of training facilities, the ergonomic integrity of workstation design, ambient environmental conditions, and the demands of daily commuting. When workplace facilities are substandard, such as the provision of improper or broken furniture, workstations with sharp corners, or chairs and tables that fail to accommodate the anthropometric diversity of users, teaching professionals are at heightened risk for musculoskeletal discomfort and chronic pain. Similarly, thermal discomfort, resulting from environments that are excessively hot or cold, and insufficient practical amenities further compound these risks, undermining both physical health and psychological well-being [21]. Extended training hours, particularly when coupled with inadequate rest periods, exacerbate fatigue and reduce teaching professionals’ capacity to recover from daily stressors, thereby increasing susceptibility to both WMSDs and psychological distress. Research underscores that poor workstation design and a lack of ergonomic consideration are strongly associated with the development of lower back pain and neck pain among teaching professionals [22].

Organisational factors constitute a pivotal independent variable influencing the health and well-being of teaching professionals, extending well beyond the immediate physical work environment. These factors encompass a range of systemic challenges, including excessive expectations from management, punitive repercussions for perceived shortcomings, insufficient administrative support, a lack of understanding of teaching professionals’ unique needs, inadequate access to essential resources, and escalating workloads. Such conditions disrupt work–life balance and contribute to the uneven distribution of responsibilities, which have been specifically linked to the onset of WMSDs among school teaching professionals in Malaysia [5]. One of the studies confirms that high WMSDs among teaching professionals affect their jobs, result in absenteeism and reduced productivity, and can lead to high medical costs [23].

Socioeconomic factors, constituting the fourth independent variable, encompass a broad spectrum of social and economic determinants that exert profound influence on the physical and psychological well-being of teaching professionals. These factors are not limited to conventional social and economic status but extend to include medical emergencies within a family unit, peer pressure, and broader national or global crises such as pandemics, wars, or natural disasters. Such events can precipitate acute stressors that disrupt both professional and personal domains, thereby amplifying the risk of mental and physical strain among teaching professionals [24]. Job stress may impact social life and create negative emotions affecting teaching professionals [25]. The COVID-19 pandemic has served as a salient example of how sudden and large-scale disruptions can compound these challenges. The abrupt transition from traditional face-to-face instruction to online teaching modalities has placed unprecedented demands on teaching professionals worldwide. Teaching professionals lacking proficiency in Information and Communication Technologies (ICT) tools have encountered additional psychological stressors, as well as musculoskeletal discomfort resulting from prolonged use of unfamiliar digital platforms [26]. 

External stakeholder factors, constituting the fifth independent variable in the aetiology of work-related stress among academic professionals, encompass a range of pressures and challenges emanating from the broader educational ecosystem. Among these, the teacher-student ratio has been identified as a critical determinant of occupational stress, with elevated ratios correlating strongly with increased workload, diminished capacity for individual student engagement, and heightened difficulty in managing classroom behaviour and discipline [19,27]. Additional stressors include institutional demands for securing external funding and the pressures associated with maintaining a positive public image, both of which can exacerbate job insecurity and workload intensity [19]. Student-related challenges, such as bullying and disruptive behaviours, further compound these stressors, as teaching professionals may struggle to maintain classroom order and foster a supportive learning environment, particularly in overcrowded or under-resourced settings [19]. The transition to online teaching modalities, accelerated by the COVID-19 pandemic, has introduced new dimensions of stress, anxiety, and depression for teaching professionals, as they navigate unfamiliar technologies, increased administrative burdens, and the complexities of remote student engagement [26]. The nature of student-teacher relationships and the attitudes of students at different grade levels are also pivotal, with research indicating that these relational dynamics are closely linked to various dimensions of teaching professionals’ burnout and job satisfaction [28].

Even before the COVID-19 pandemic, teaching professionals were already at heightened risk for WMSDs and psychological stress due to factors such as poor posture, repetitive tasks, and limited control over their work environment, which often led to physical ailments like back pain, carpal tunnel syndrome, and joint stiffness, as well as mental health struggles including anxiety and burnout [10,11]. The chronic demands of classroom management, meeting academic expectations, and striving to maintain a healthy work–life balance further compounded these challenges, resulting in increased psychological strain and emotional exhaustion [29]. This combination of physical and psychological stressors not only undermined teaching professionals’ health and well-being but also diminished their capacity to deliver effective instruction and engage positively with students.

The COVID-19 pandemic significantly exacerbated the existing challenges faced by teaching professionals, as the abrupt transition to online instruction led to prolonged periods of computer use, inadequate ergonomic conditions, and reduced physical activity, all of which heightened the risk of WMSDs, particularly for teaching professionals lacking suitable home-office environments [30]. Concurrently, the isolation inherent in remote work, combined with the necessity to adapt to new digital platforms and teaching methodologies rapidly, intensified psychological stress and emotional strain. Many teaching professionals reported increased levels of exhaustion, burnout, and anxiety as they struggled to maintain student engagement and pedagogical effectiveness within virtual classrooms, underscoring the profound and multifaceted impact of the pandemic on both the physical and mental health of teaching professionals [31,32].

In the post-pandemic period, numerous teaching professionals continue to confront enduring WMSDs and psychological stress, as the hybrid teaching model combining in-person and virtual instruction has yet to relieve the physical and emotional challenges teaching professionals face fully. Even as many have adjusted to online platforms, the persistent demands for delivering high-quality education and the uncertainties surrounding future teaching modalities contribute to ongoing physical discomfort and mental exhaustion [5]. These factors, compounded by workload pressures and insufficient support, sustain high risks for both WMSDs and psychological distress among teaching professionals, underscoring the critical need for comprehensive strategies to address these issues and promote teaching professionals’ well-being in contemporary education systems.

The causing variables, workplace WMSDs and psychological stress, are not only closely associated but also influence each other in a bidirectional manner. It means that experiencing WMSDs can contribute to increased psychological stress, and, conversely, high psychological stress can exacerbate or even precipitate musculoskeletal symptoms [33]. To fully understand the consequences of these intertwined conditions, the review will further examine four specific outcome variables: impaired job performance, short-term health impairment (such as increased sick leave or reduced work ability), job dissatisfaction, and negative impacts on social and family life. By monitoring these outcomes, the research aims to capture the multifaceted ways in which WMSDs and psychological stress affect teaching professionals, both within and beyond the workplace.

The framework presented in Table 2 offers a comprehensive synthesis of the multifactorial determinants influencing the prevalence of WMSDs and psychological stress among teaching professionals. By adopting a multi-dimensional analytical lens that encompasses organisational, socioeconomic, external stakeholder, human, and workplace dimensions, this framework elucidates the intricate and dynamic interplay of factors shaping teaching professionals’ physical and psychological well-being. Notably, organisational determinants such as heightened expectations, insufficient managerial support, and excessive workloads emerge as foundational contributors to both physical strain and emotional distress. The impact of these organisational pressures is further compounded by rapid internal curriculum revisions and policy changes, which often occur without adequate resources or lead time for adaptation.

Socioeconomic variables, including financial insecurity and personal or familial stressors, introduce additional layers of complexity that can exacerbate teaching professionals’ vulnerability to health impairments. Concurrently, external stakeholder factors, particularly the relational dynamics between teaching professionals and students, as well as the challenges posed by disruptive student behaviour and elevated student-teacher ratios, amplify occupational stress and complicate classroom management. Immediate workplace causes, such as suboptimal ergonomic conditions, extended training hours, and ineffective management of pedagogical practices, further intensify the physical and mental demands placed on teaching professionals.

Collectively, these interconnected factors foster a work environment in which musculoskeletal disorders and psychological stress are pervasive, highlighting the necessity for holistic, evidence-based interventions. Addressing these challenges mandates a multi-pronged strategy that includes targeted organisational reforms, enhancements to working conditions, and the cultivation of robust support networks involving both educational institutions and the broader community. Such an approach is essential for safeguarding the well-being of teaching professionals and ensuring the sustainability of high-quality educational systems.

## 4. Mechanism of Different Factors Contributing to WMSD and Psychological Stress

This study stands out for its dedicated focus on teaching professionals for adult learners, a demographic often underrepresented in occupational health research. Unlike conventional studies that concentrate on school or university teachers, this review addresses the unique occupational challenges faced by teaching professionals for adult learners, such as managing diverse learner profiles, navigating accreditation pressures, and adapting to hybrid or non-traditional teaching environments. These teaching professionals operate in settings that demand a high level of physical, cognitive, and emotional engagement, yet their health risks are rarely examined in a comprehensive manner.

The study’s second major contribution is the development of a multifactorial framework that integrates five key domains: human, workplace, organisational, socioeconomic, and external stakeholder factors. This framework moves beyond isolated risk factors to capture the complex, interdependent nature of WMSDs and psychological stress. By doing so, it provides a more holistic understanding of how these conditions arise and interact, particularly emphasizing the bidirectional relationship where physical pain exacerbates psychological stress and vice versa [64,65]. Recent findings have shown that musculoskeletal pain and psychological burden among teaching professionals are strongly linked to workload intensity, prolonged standing or sitting, and insufficient psychosocial support, highlighting the urgent need for integrated ergonomic and mental health interventions. This comprehensive approach advances existing models by aligning with contemporary occupational safety research and addressing the evolving demands of modern teaching environments.

Furthermore, the study innovatively synthesises established models into a unified conceptual structure. This integration allows for a more in-depth analysis of teaching professionals’ well-being and offers a foundation for future empirical validation. Recent research has demonstrated the value of combining psychosocial and ergonomic perspectives to understand the interplay between occupational demands, effort–reward imbalance, and health outcomes in educational settings [8,9]. The framework not only informs targeted interventions but also supports policy development aimed at improving occupational health outcomes for adult teaching professionals, making it a valuable tool for researchers, institutions, and policymakers alike.

The five interconnected factors—human, workplace, organisational, socioeconomic, and external stakeholder—form a complex, adaptive system influencing WMSDs and psychosocial stress among teaching professionals for adult learners, as shown in Table 2. The integration of the accident causal models highlights how linear causal chains such as poor posture, extended teaching hours, and heavy workload coexist with non-linear feedback loops between human, organisational, and contextual elements. Physical and psychological health are reciprocally linked, where musculoskeletal discomfort heightens emotional fatigue and, in turn, further exacerbates poor posture and reduced tolerance [5,31,47]. These interactions illustrate how both immediate ergonomic factors and systemic organisational pressures shape occupational outcomes in an interdependent manner.

Within this systemic structure, workplace and organisational factors serve as amplifiers of risk. Inadequate ergonomic design, long teaching hours, and poor ventilation [22,49] interact dynamically with organisational shortcomings such as excessive expectations, weak institutional support, and rapid curriculum changes [10,40,41]. From a linear perspective, these are direct causal stressors; however, non-linear theory explains how feedback loops such as accumulated fatigue leading to reduced performance and further managerial pressure sustain a reinforcing cycle of strain. The hybrid learning and digital transition have further intensified this complexity, linking cognitive overload to both physical discomfort and mental exhaustion [42,56].

Socioeconomic and external stakeholder influences add further non-linear variability, creating emergent patterns of stress and health decline that cannot be explained through single-cause models. Financial strain, job insecurity, and family obligations [16,30] intersect with disruptive student behaviour and institutional accountability mechanisms [61,63] to produce overlapping feedback effects across personal, organisational, and societal levels. These interdependencies embody the systems-based nature of occupational risk in adult education, where psychological distress and WMSDs evolve through constant adaptation and interaction among variables. Addressing these challenges requires interventions that simultaneously target ergonomic design, organisational justice, and psychosocial support to disrupt reinforcing feedback cycles and promote long-term well-being among teaching professionals.

The integration of prevalence data and contributing factors of WMSDs and psychological stress among teaching professionals from both developed and developing countries requires a nuanced, context-sensitive approach. While the core occupational stressors—such as poor ergonomics, prolonged static postures, and high workload—are universally present, their intensity and impact vary significantly across socioeconomic settings. In developed countries, teaching professionals often face technologically driven stressors, including digital fatigue, administrative overload, and performance pressure, which are somewhat mitigated by institutional support systems and access to ergonomic infrastructure and mental health services [9]. In contrast, teaching professionals in developing and underdeveloped regions frequently encounter compounded challenges such as inadequate classroom infrastructure, overcrowded student-teacher ratios, limited access to occupational health services, and socioeconomic instability [66]. These disparities underscore the need for a harmonised framework that identifies universal risk domains—such as workload intensity, low job control, and poor posture—while also accounting for region-specific stressors like lack of institutional support and policy enforcement. For example, while neck and lower back pain are common across all contexts, their root causes may differ: digital overuse in high-income settings versus poor furniture and long commutes in low-resource environments [67].

**Figure 4:**
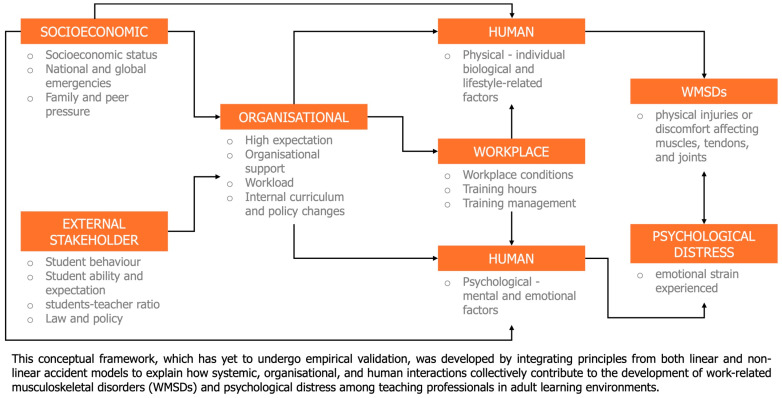
Multifactorial framework contributors to WMSDs and psychological distress in teaching professionals for adult learners.

In sum, the cumulative effect of these interrelated factors is a high-pressure environment that challenges the sustainability of adult education, particularly for its teaching professionals. The mechanisms through which these factors interact—feedback loops, compounding stressors, and institutional inertia—require a systemic response that is tailored to the realities of the teaching professionals’ role. Solutions must be holistic, addressing not only the immediate symptoms such as workload or classroom behaviour, but also the underlying structural and relational dynamics. It includes redesigning workplace environments for physical comfort, providing mental health support that reflects the emotional demands of teaching, fostering inclusive and participatory organisational cultures, and educating stakeholders—especially policymakers and institutional leaders—on the realities and needs of adult teaching professionals. Only through such integrated interventions can the profession be safeguarded and empowered to meet the evolving demands of adult education. Refer to Figure 4 for the Framework of multifactorial causes for WMSDs and psychological stress.

This framework is grounded in contemporary systems-based accident theories that view occupational health outcomes as emergent results of complex, interdependent interactions within socio-technical systems. It draws primarily from the non-linear System-Theoretic Accident Model and Processes (STAMP) proposed by Leveson, which conceptualises risk as a dynamic control problem within hierarchical structures, and from Reason’s Organizational Accident Model, which highlights latent and active failures across organisational layers. Complementing these, Rasmussen’s Risk Management Framework introduces the concept of multilevel interactions among policy, management, and operational domains [68]. By combining these theoretical perspectives, the framework captures how structural, psychosocial, and human elements in educational environments interact to influence work-related musculoskeletal disorders (WMSDs) and psychological distress among trainers of adult learners. Recent studies confirm that psychosocial factors such as job control, support, and workload are significantly associated with MSDs, and that teaching professionals are increasingly affected by low back pain and WMSDs due to prolonged static postures and cognitive demands [8].

The framework integrates socioeconomic, external stakeholder, organisational, workplace, and human factors to demonstrate how systemic and personal determinants converge in shaping trainers’ physical and mental health. Socioeconomic and stakeholder conditions form the contextual backdrop that influences institutional culture, workload, and resource allocation. These organisational conditions, in turn, affect workplace design, training schedules, and managerial support, which directly shape the physical and psychological demands placed on trainers. The human component, both physical and psychological, represents the interface where cumulative pressures manifest as WMSDs and emotional strain [69]. This interconnected structure illustrates a feedback loop: adverse outcomes can weaken organisational performance and feedback into higher-level policy decisions, emphasising the non-linear, adaptive nature of risk development in teaching environments.

Policies (being a significant underlying solutions) could require regular ergonomic risk assessments, confidential psychosocial screening, and a real-time reporting system for teaching professionals to flag physical or emotional strain. These measures should feed into continuous policy review cycles that monitor WMSD rates, sickness absenteeism, and feedback from reflective surveys to inform timely adjustments in workload, training design, and institutional support.

Although this model has not yet been empirically tested, its reliability is strengthened by its theoretical derivation from well-established, validated systemic models in safety and ergonomics research. Each layer of the framework corresponds to constructs supported by extensive evidence in occupational health literature—such as psychosocial stress theory, ergonomic risk assessment, and systems ergonomics. Its multilevel structure enhances explanatory depth and provides flexibility for quantitative or qualitative adaptation across diverse educational settings. Consequently, the framework offers a theoretically sound and comprehensive basis for future empirical validation, enabling researchers to test causal relationships and intervention pathways linking organisational design, psychosocial dynamics, and trainer well-being.

Future interventions addressing WMSDs and psychosocial stress among teaching professionals for adult learners should adopt an integrated, system-based approach that combines ergonomic design, organisational reform, and psychosocial support. Preventive strategies should prioritise redesigning teaching environments to reduce static postures and repetitive strain, supported by structured rest periods and ergonomic training. At the organisational level, improved workload management, enhanced communication channels, and equitable recognition systems are essential to mitigate psychosocial strain. It is also suggested that policies incorporate continuous risk monitoring using both quantitative and qualitative feedback to capture dynamic human–system interactions. Strengthening institutional awareness and embedding preventive ergonomics and mental health promotion within professional training programmes would ensure more sustainable well-being outcomes for teaching professionals in adult education settings.

## 5. Conclusions

The understanding of work-related risks among teaching professionals requires a conceptual transition from linear to non-linear perspectives of occupational causation. Linear reasoning has long dominated traditional approaches by emphasising direct and sequential cause-and-effect relationships, which often simplify complex realities into isolated variables. Although this approach is useful for identifying individual risk factors such as workload, posture, or stress, it offers limited insight into the interconnected nature of teaching environments. The diverse challenges faced by teaching professionals, particularly in professional training contexts, demonstrate that occupational health outcomes rarely result from single sources but instead emerge from interacting systems.

A non-linear perspective provides a more comprehensive view by acknowledging that multiple human, organisational, and environmental factors interact simultaneously and continuously. This view aligns more closely with the lived experiences of teaching professionals, where evolving curricula, technological integration, and diverse learner needs create dynamic and adaptive work conditions.

The most significant contribution of the proposed framework is its integration of linear clarity with non-linear adaptability. By linking measurable determinants of risk with complex systemic interactions, the framework establishes a balanced foundation for understanding and managing the multifaceted physical and psychological demands faced by teaching professionals in professional training environments. The proposed framework is distinct from existing models because it combines both sequential cause–effect logic and emergent system behaviour into a single structure, enabling individual, organisational, and contextual factors to be understood simultaneously as direct risk pathways and as dynamic feedback loops.

The authors recommend that future interventions addressing WMSDs and psychosocial stress among teaching professionals for adult learners adopt an integrated, system-based approach that combines ergonomic design, organisational reform, and psychosocial support. Preventive strategies should prioritise redesigning teaching environments to reduce static postures and repetitive strain, supported by structured rest periods and ergonomic training. At the organisational level, improved workload management, enhanced communication channels, and equitable recognition systems are essential to mitigate psychosocial strain. The authors further suggest that policies incorporate continuous risk monitoring using both quantitative and qualitative feedback to capture dynamic human–system interactions. Strengthening institutional awareness and embedding preventive ergonomics and mental health promotion within professional training programmes would ensure more sustainable well-being outcomes for teaching professionals in adult education settings.

This review is constrained by its reliance on secondary literature and the absence of a formal quality appraisal, and the proposed framework remains theoretical without empirical testing. Its applicability may also vary across different adult education contexts. Future work should validate the framework through empirical studies, assess its relevance across diverse settings, and refine its components using real-world data to strengthen its practical and policy utility.

## Figures and Tables

**Table 1:** Detailed Search Strategy.

Database	Date of Search	Search Terms (Boolean Combinations)	Filters/Limiters	Records Retrieved	Records Included
ScienceDirect	October 23–June 25	(“Workplace Musculo-skeletal Disorder AND psychological stress”) AND (“School teacher”) OR (“teaching professionals”)	English, peer-reviewed articles	18	8
PubMed	October 23–June 25	(“musculoskeletal disorders” OR “WMSDs”) AND (“teaching professionals”)	English, peer-reviewed articles	28	18
Scopus	October 23–June 25	(“psychological stress”) AND (“adult learners” OR “adult education”)	English, full-text available	12	1
Web of Science	October 23–June 25	(“occupational health” OR “workplace health”) AND (“ergonomics” OR “job control”)	English, peer-reviewed articles	32	30
Google Scholar	October 23–June 25	Combination of above terms, phrase searching, truncation applied	English	78	19

**Table 2:** Framework for causes of WMSDs and Psychological Stress.

Key Factor (1st Layer)	Subfactor (2nd Layer)	Subfactor (3rd Layer)	Subfactor (4th Layer)	References
Human factor	Physical	Age	A reduction in strength and endurance	[5,18,30,34,35,36,37,38,39]
		Height	Affecting reach	
		Weight	BMI over borderline	
		Sex	Gender impact	
		Nutrition	Poor food choices and a lack of sufficient nutrition for physical and mental growth	
		Physical exercise	Lack of physical exercise, sedentary lifestyle	
		Genetics	Parents and family members having related diseases	
		Health ailments	Past medical records and surgeries	
		Posture	Poor work posture	
	Psychological	Attitude	A poor attitude such as excessive confidence, stubbornness, arrogance, selfishness, showing off, lack of focus, violation of proper procedure due to time or work pressure;	[7,15,26,31,40,41,42,43,44]
		Experience	Lack of skills and knowledge; complicated procedures;	
		Self-confidence and Realisation	Lagging confidence; lack of motivation; poor communication and coordination	
		Low mood and tolerance level	Distressed personality, loneliness, difficulty in teaching online classes	
Workplace Factor	Workplace conditions	Resources	Lack of adequate resources	[22,45,46,47,48]
		Ventilation system	Poor ventilation system (too cold or too hot environment)	
		Workstation design	Tables and chairs are not comfortable or damaged	
	Training hours	Long training hours	Long training hours lead to physical and mental stress, also resulting in voice disorders	[49,50]
		Travel distance to commute to a training centre	Long distances and training at different locations can cause tiredness and mental stress when reaching the training centre on time	
	Training management	Poor practical and training facilities	Practical items missing or not arranged properly; training books or assessment documents are inadequate	[32]
		Poor facilities in a classroom	Projector malfunctions; computers not working, or cable misaligned	
		Class methods	Conducting both online and face-to-face classes	
		Coping with curriculum changes	Lack of time to get accustomed to new changes in the curriculum, or errors and mistakes in the new curriculum	
Organisational factor	Expectation	High expectations from teaching professionals	Lack of physical strength and mental distress to meet expectations, Unrealistic expectation Unable to cope	[40,41,47]
	Organisational Support	Lack of organisational support	Management’s lack of support and understanding affects the teaching professionals’ morale	[41,45,51]
			Lacking trust	
			Lack of communication and participation	
			Unheard expectations for teaching professionals	
	Workload	Extra workload	Work overload and coping with increased responsibility	[5,14,41,45,47,52]
		New work task	Unfamiliar with new tasks	
		Time	Time pressure to complete a task in a short duration	
		Salary	The workload doesn’t match the salary paid	
	Internal curriculum and policy changes	Undue pressure due to changes in curriculum and poor policies	Extra pressure to cope with new changes in the course syllabus and understand a new concept	[10,51]
			Curriculum changes were not communicated in advance	
			Policy not implemented effectively	
			No review or feedback is taken from teaching professionals before making changes	
			Poor Communication	
Socioeconomic factor	Socioeconomic status	Family and social commitments and expenditure	As teaching professionals need to earn more money to meet commitments, they put in extra time and effort and ignore their body and mental limitations	[4,16,17,26,30,44,53]
		A family medical emergency, such as an accident or death among family, relatives, or close friends	Experience emotional distress and feel low	
			Unable to concentrate due to family emergencies	
			Dilemma for replacement	
	Country or Global Emergency	Pandemic situation	COVID-19 pandemic impact trainer	[16,54,55,56]
			Change in the mode of classes and assessment via online	
	Family and peer pressure	Lack of family and peer support	Family pressure to spend more time	[25,57,58]
			Unable to participate in social gatherings with close friends or relatives	
			Lack of sufficient financial support	
External stakeholder factors	Student behaviour	Student misbehaviour towards trainers		[10,59,60]
		Lack of respect and rude behaviour		
		Bullying others and teaching professionals		
	Student ability and expectation	Students’ poor ability to grasp, understand and comprehend		[33,61]
		Low or high expectations from the student		
		An exam-centric mindset of students		
	Students complaining to either the training institute or the authorities	Unfair complaints about teaching professionals to the respective institute by students		[62]
		Report concerns to the relevant regulatory authorities and oversight bodies		
	Students-teacher ratio	Number of students in one class	Too many students in one class	[5,27]
			Students get distracted easily	
			Difficult to manage	
	Law and policy	Lack of funding, new enforcement	Impact on how the course can run, changes in policy at the government level	[33,63]

## Data Availability

No new data were created or analysed in this study.
